# Supplementary material for: Efficient indexing and querying of annotations in a pangenome graph
Source: bioRxiv. 2024 Oct 15:2024.10.12.618009. Preprint. [Version 1] doi: 10.1101/2024.10.12.618009 (PMC11507721; doi:10.1101/2024.10.12.618009)
Supplement: 1 [file NIHPP2024.10.12.618009V1-supplement-1.pdf]

bioRxiv preprint doi: https://doi.org/10.1101/2024.10.12.618009; this version posted October 15, 2024. The copyright holder for this preprint (which was not certified by peer review) is the author/funder, who has granted bioRxiv a license to display the preprint in perpetuity. It is made available under aCC-BY 4.0 International license.

Supplementary material

Table 3: Compute resources used for the analysis of the functional datasets and production of the indexed coverage tracks. The *coverage track* step includes computing coverage profile on the pangenome using `vg pack`, making coverage tracks using a python script, and compressing the output GAF with `gzip`.

Dataset	reads (M)	read mapping	coverage track	sorting + compressing + indexing
Breast epithelium	193.6	8.9 CPU-H (54 GiB)	3 CPU-H (109 GiB)	0.3 CPU-H (1 GiB)
Gastrocnemius medialis	98.8	4.8 CPU-H (54 GiB)	2.6 CPU-H (99 GiB)	0.3 CPU-H (1 GiB)
Gastroesophageal sphincter	168.5	7.3 CPU-H (54 GiB)	3 CPU-H (108 GiB)	0.2 CPU-H (1 GiB)
Peyer's patch	145.3	8 CPU-H (54 GiB)	3 CPU-H (104 GiB)	0.2 CPU-H (1 GiB)
Sigmoid colon	173.5	8.2 CPU-H (54 GiB)	3 CPU-H (106 GiB)	0.3 CPU-H (1 GiB)
Spleen	157.2	7.6 CPU-H (54 GiB)	3 CPU-H (104 GiB)	0.2 CPU-H (1 GiB)
Thyroid gland	91.4	4.6 CPU-H (54 GiB)	2.5 CPU-H (94 GiB)	0.1 CPU-H (1 GiB)
